# On the Dynamics of a Novel Liquid-Coupled Piezoelectric Micromachined Ultrasonic Transducer Designed to Have a Reduced Resonant Frequency and Enhanced Ultrasonic Reception Capabilities

**DOI:** 10.3390/mi15101210

**Published:** 2024-09-29

**Authors:** Stephen Sammut, Edward Gatt, Ruben P. Borg

**Affiliations:** 1 Institute of Engineering and Transport, Malta College of Arts Science and Technology, 9032 Paola, Malta; 2 Faculty of ICT, University of Malta, 2080 Msida, Malta; edward.gatt@um.edu.mt; 3 Faculty for the Built Environment, University of Malta, 2080 Msida, Malta; ruben.p.borg@um.edu.mt

**Keywords:** PMUT, ultrasonic, diaphragm, resonance, embedded sensors

## Abstract

This paper introduces a novel design for a liquid-deployed Piezoelectric Micromachined Ultrasonic Transducer (PMUT). This design was specifically developed to resonate at a lower ultrasonic frequency than a PMUT with a circular, fully clamped diaphragm with the same diameter. Furthermore, the novel design was also optimised to enhance its ultrasonic radiation reception capabilities. These parametric enhancements were necessary to develop a PMUT device that could form part of an eventual microscale sensory device used for the Structural Health Monitoring (SHM) of reinforced concrete (RC) structures. Through these two enhancements, an eventual microscale sensor can be made smaller, thus taking up a smaller die footprint and also be able to be deployed further apart from each other. Eventually, this would reduce the developed distributed sensor system’s cost. The innovative design employed a configuration where the diaphragm was only pinned at particular points along its circumference. This paper presents results from Finite Element Modelling (FEM), as well as experimental work that was conducted to develop and test this novel PMUT. The experimental work presented involved both laser vibrometry and ultrasonic radiation lab work. The results show that when compared to a clamped diaphragm design, the novel device managed to achieve the required reduction in resonant frequency and presented an enhanced sensitivity to incoming ultrasonic radiation.

## 1. Introduction

Reinforced concrete is one of the most widely used structural materials in the world. Concrete’s structural and chemical integrity may be undermined by chemical or physical factors that lead to the initiation of structural degradation. It is for this reason that structures made of reinforced concrete require what is known as Structural Health Monitoring (SHM). Through an effective SHM system, the structures can be monitored and action can be taken if the presence of degradation factors is determined. One such degradation factor is chloride ion ingress, which causes the initiation of rebar corrosion. Steel rebar does not normally corrode when it is in contact with the concrete’s alkaline pore solution.

However, chloride ions, which access the concrete’s internal rebar structure through the pore solution, attack the rebar’s passivation layer and initiate corrosion [1,2,3]. When the rebar corrodes, it expands and damages the surrounding concrete structure. Since chloride ion diffusion fronts are slow moving, the ideal way of detecting *Cl*^−^ ions within a structure is through the deployment of a distributed sensor network [4,5,6].

**Figure 1:**
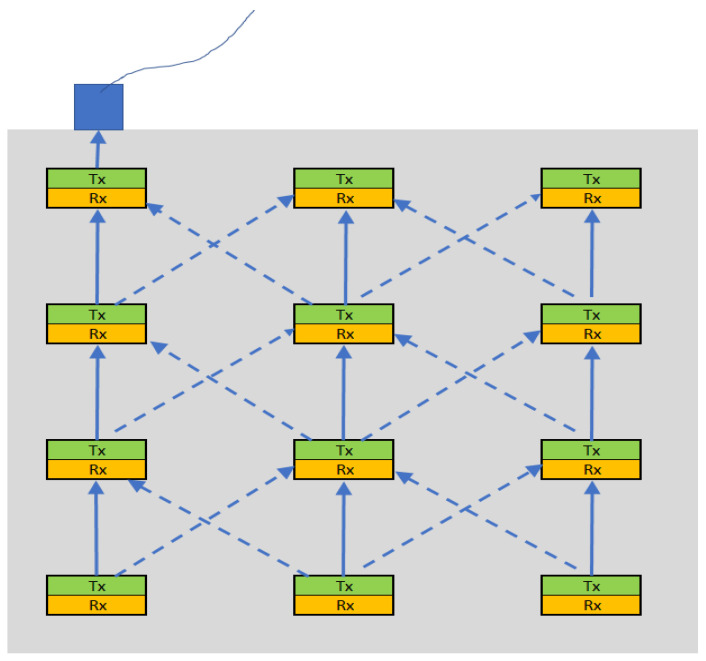
A concept of a distributed sensory system deployed in RC [7].

The devices forming part of this network of sensors are distributed throughout the structure at the construction stage, and they spend years in a dormant state only to transmit a signal when *Cl*^−^ ions are detected. This data channel can be established through the use of an ultrasonic radiation transmission path. The conceptual diagram is shown in Figure 1, which shows a model of a distributed sensor system embedded in reinforced concrete.

The grey shaded area in Figure 1 symbolises the reinforced concrete structure. The sensory devices are distributed throughout the structure with each device having a transmitting part (green) and a receiving part (yellow). The wired device shown in blue is the receiving device positioned at the surface of the structure. Through the surface device, engineers can receive real-time notifications if any of the embedded devices detect adverse structural issues within the RC structure. This application works using a one-way mode, where the sensory devices always transmit data towards the surface device when they detect off-limit parameters. With reference to Figure 1, the transmitting components (Txs) will be expected to operate only in transmit mode, while the receiving components (Rxs) will work only in reception mode. Since the particular components will not be working as transceivers, they can be designed as optimised ultrasonic radiation receivers or transmitters.

PMUTs can serve as a means for distributed sensors within concrete structures to transmit and receive data between each other when deployed inside the structure [5,6]. Depending on whether the PMUT is excited electrically or acoustically, it can serve as a transmitter or a receiver of ultrasonic radiation.

To acoustically couple with the solid RC structure, the PMUT requires a liquid coupling fluid. Gases normally present low values of acoustic impedance, with air having an acoustic impedance of 420 kg m^−2^s^−1^. The acoustic impedance of gases is much lower than that of solids and liquids. This factor leads to the reflection and loss of acoustic energy rather than its transmission into the solid if a gaseous coupling fluid is employed [8]. For this reason, liquid coupling was used for this project.

Experimental studies reviewed in the literature suggest that, for optimal propagation of ultrasonic radiation through RC, the frequency of the ultrasonic radiation should ideally be below 100 kHz [8]. This frequency is significantly lower than the resonant frequency range at which most liquid-deployed PMUT devices reviewed in the literature operate [9,10,11,12].

**Figure 2:**
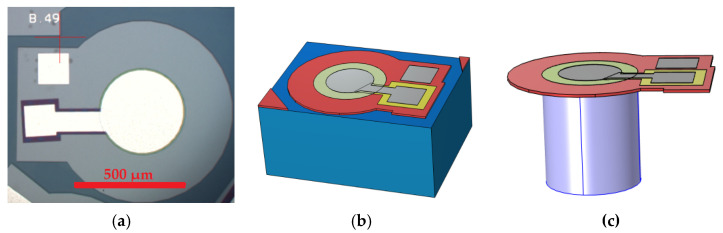
Front side micrograph of (**a**) a circular, fully clamped diaphragm PMUT device which was used as control device; Finite element model showing (**b**) the complete device and (**c**) the silicon PMUT substrate removed to show the fluid column which resides inside the cavity [7].

The current state-of-the-art PMUTs, as reviewed in the academic literature, are of the circular clamped diaphragm design, which is shown in the micrograph in Figure 2a. This figure presents a frontal micrograph of a circular, clamped diaphragm, sealed-cavity PMUT.

In the figure, white structures can be observed; these are the aluminium pad and electrode metal layer structures. The oxide layer is shown in purple, while the piezoelectric layer is hidden under the metal layer. Beneath this piezoelectric layer is the doped silicon diaphragm structure. The piezoelectric layer is therefore situated between two electrodes, namely the top aluminium electrode and the bottom doped silicon electrode. These electrodes can either excite the piezoelectric layer, causing the diaphragm to deflect, or detect a voltage when the piezoelectric layer is displaced along with the diaphragm due to incoming acoustic radiation. As can be seen in the micrograph, the diaphragm in the control device is fully clamped to the substrate around its entire circumference. This structure is further illustrated in Figure 2b,c which show details of the finite element model built to simulate the PMUT before it was constructed.

As will be explained in detail below, for clamped diaphragm devices, the resonant frequency is directly related to the diaphragm’s diameter. In view of the relatively low frequency being used in this field, the diaphragm size would need to be relatively large. This presents significant space challenges on a die’s footprint, particularly when deployed in an array configuration, which typically consists of hundreds or even thousands of PMUTs [13].

Reducing the PMUT diameter would therefore lead to a substantial decrease in the overall size of a microscale device, aligning with the design objectives outlined in this paper. Furthermore, since the transmitting and receiving devices for this project will be specialised, the novel device presented in this paper was optimised for the reception of ultrasonic radiation.

The design of the novel device was based on an unclamped diaphragm structure pinned at specific points rather than along its entire circumference. Previous studies indicated that a reduction in resonant frequency could be achieved with a pinned diaphragm configuration [14]. Furthermore, the literature also indicated that a pinned boundary configuration does allow for a larger amplitudes of deformation when compared with a clamped diaphragm setup. This results in higher electromechanical coupling [15]. However, this prior research only provided evidence for pinned diaphragm PMUTs which were designed to operate with air coupling.

Given that liquids are denser than gases and present a different fluid dynamic scenario, it was necessary to completely develop and validate a new pinned diaphragm PMUT design capable of operating in liquids. The novel design thus needed to be specifically engineered to operate at a resonant frequency within the 70 to 110 kHz range in liquid coupling fluids. Finite Element and experimental processes were conducted to determine and confirm the improvements imparted by the novel design.

## 2. The Design Process

### 2.1. The State of the Art

The authors had previously published work setting out an equation that relates the PMUT diaphragm’s diameter with its resonant frequency. This relationship holds for isopropanol deployed, circular clamped diaphragm PMUTs having trench diameters ranging between 550 µm and 2000 µm [16]. This relationship was established through laser vibrometry.

**Figure 3:**
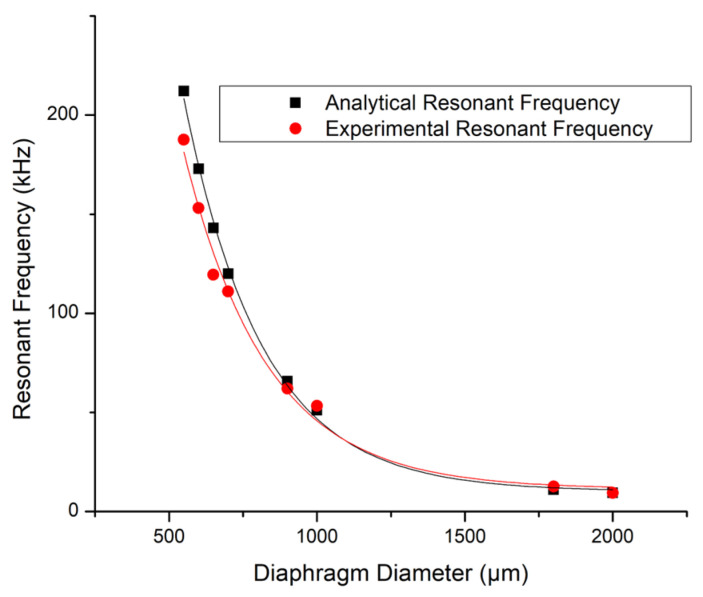
Graph showing the relationship between the experimental and analytical Resonant Frequency values versus the PMUT’s Diaphragm Diameter.

In this paper, the authors enhanced the accuracy of this equation further by adding additional resonant frequency values including values achieved by a 1800 µm diameter PMUT. The final updated resonant frequency versus PMUT diameter curve is being presented in Figure 3. The analytical resonant frequency values also shown in Figure 3 were calculated using equations which were similarly presented in the author’s prior paper [16].

This best-fit curve for the experimental values presented in Figure 3 is expressed using Equation (1).

(1) $$f=3\times 10^{8}D^{-2.284}$$

where

*f* is the resonant frequency [kHz]

*D* is the PMUT diaphragm’s diameter [µm]

This equation expresses the PMUT’s resonant frequency in terms of its diaphragm’s diameter. It evidences the fact that for a clamped diaphragm PMUT, to achieve lower resonant frequencies, one requires an increase in the diameter of the PMUT’s diaphragm.

As outlined by Equation (1), achieving the required low resonant frequencies would necessitate a clamped diaphragm PMUT with a relatively large cavity diameter of over 750 µm.

### 2.2. The Device Concept

**Figure 4:**
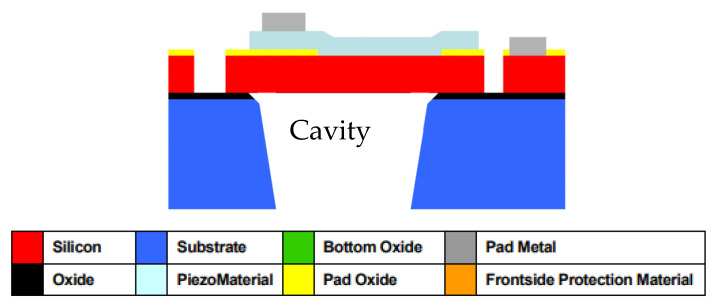
Section through a PMUT created using the PiezoMUMPS^TM^ process [17].

The devices were designed and built using the PiezoMUMPS^TM^ fabrication process. Figure 4 illustrates a section through a circular clamped diaphragm PMUT, constructed using the PiezoMUMPS^TM^ process. This figure also highlights the cavity and diaphragm structures, on which the PiezoMUMPS^TM^ fabrication process deposits a 0.5 µm thick AlN piezoelectric layer; this layer deforms when electrically excited, and generates an electric voltage when acoustically stimulated.

In the experimental procedures described in this paper, isopropanol was selected as the coupling fluid due to its ability to provide an inert environment. This choice was made after deionized water was observed to have a deteriorative effect on the PMUT structure, particularly its metal layers. Lab tests with isopropanol showed that it did not react in any way with the PMUT structures.

**Figure 5:**
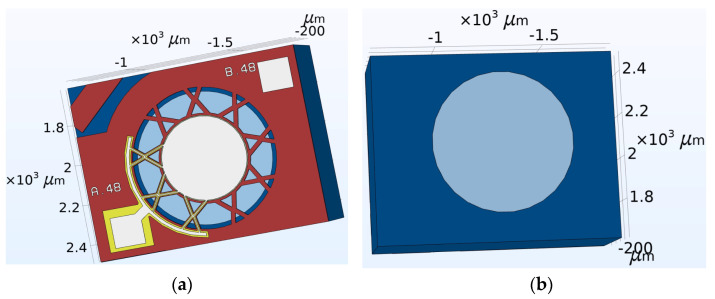
The novel pinned diaphragm PMUT device showing (**a**) the front side view and (**b**) backside view showing the cavity opening [7].

The novel design concept involved a centrally suspended diaphragm structure supported by eighteen arms which were organised in a cross formation, as illustrated in Figure 5a. The central structure contains the central electrode overlying the piezoelectric layer, which, in turn, lies over the doped silicon diaphragm. The padmetal electrode layer for the novel device had a diameter of 400 µm resulting in a 62% electrode radial coverage. This percentage electrode radial coverage was in line with the percentage electrode radial coverage used for the control device. This size similarity was important to be able to compare the results of the benchmark and novel devices.

Parametric studies, through the use of COMSOL multiphysics, were conducted to optimise the size and shape of the novel device. The back side of the device showing the cavity opening is shown in Figure 5b.

The optimised PMUT design presented in Figure 5 was achieved through the finite element process outlined in the next section.

### 2.3. The Finite Element Modelling Process

**Figure 6:**
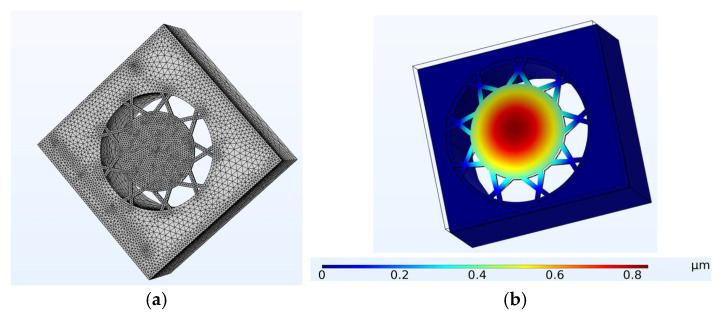
(**a**) Meshed model of the novel device showing the structural components including the suspended diaphragm. (**b**) Simulation of the diaphragm’s maximum displacement when electrically excited with a 14 V_p–p_ sine wave, indicating that peak displacement was above 0.8 µm at 77.1 kHz [7].

The software used to conduct the finite-element modelling was COMSOL Multiphysics version 6.2. Figure 6a shows the novel device’s meshed structural component. It shows the PMUT diaphragm, the suspending arms and the underlying cavity region. To resolve the model, a combination of tetrahedral and swept meshing techniques was employed. A notable challenge was the significant scale disparity, with the AlN layer being 0.5 µm thick, while the RC structure, cavity and coupling regions ranged from centimetres to metres in size.

**Figure 7:**
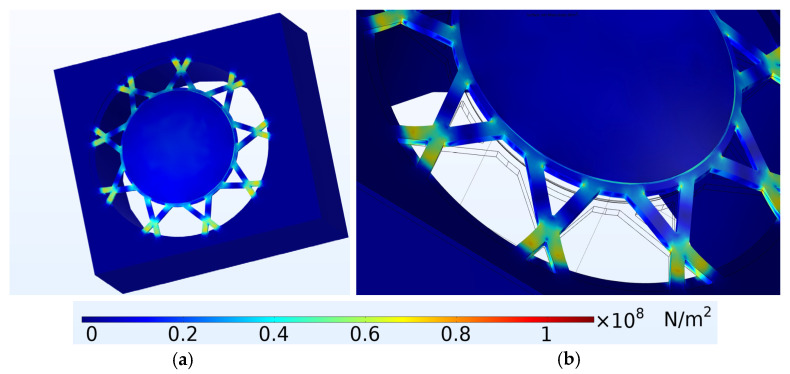
(**a**) Finite Element Modelling of the peak Von Misses stress levels when the novel device was in peak displacement. In this figure, the PMUT’s movement is magnified by a factor of one hundred; (**b**) close-up view showing the areas with the highest stress levels [7].

Apart from dynamic performance considerations, the design was also optimised to ensure that the stresses on the arms remained within acceptable limits. This was carried out through structural mechanics Finite Element Analysis, which ensured both the dynamic stability as well as the reliability of the structure. Figure 7a shows the results of the structural mechanical modelling, which was carried out to calculate the expected maximum Von Misses stress levels when the diaphragm was at the point of maximum displacement. Figure 7b shows a close-up view of the supporting arms, indicating the areas that have the highest stress levels.

The maximum stress level calculated for the novel device deployed in liquid isopropanol and with an air-filled cavity was calculated to be $1.15\times 10^{8}\;N/m^{2}$. The Young’s Modulus of the doped silicon used to produce the diaphragm in this project was calculated to be 120 GPa [7]. This indicated that the structural components would not be stressed beyond their elastic limit and, therefore, would not undergo plastic deformation. Due to this consideration, it was assured that the device would return to its original shape once the applied stresses were removed.

**Figure 8:**
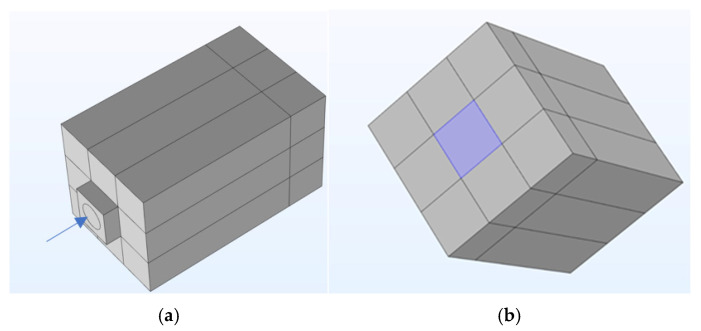
Complete FEM showing the (**a**) bottom side view with the blue arrow pointing towards the PMUT cavity and the (**b**) top view of the coupling fluid volume, highlighting the blue boundary through which the incident ultrasonic waves were introduced [7].

The structural components of the PMUT’s model were enclosed by the elements representing the fluidic regions, forming the complete model as shown in Figure 8. The lower, smaller cuboid structure marked by the blue arrow in Figure 8a represents the PMUT structural model along with the fluid in the cavity region. With reference to the same figure, the larger cuboid structure above the structure, models the coupling fluid region.

This composite FEM model was used to simulate a PMUT being excited both electrically, thus functioning as an ultrasonic transmitter, and acoustically, thereby acting as an ultrasonic receiver.

The initial FEM simulation involved modelling the behaviour of both the novel and control devices when under electrical excitation. As stated, the device was optimised for the reception of ultrasonic radiation. However, transmission FEM and experimental processes were still conducted to holistically study the novel device. Furthermore, electrical stimulation was used to test the device for reliability, as explained later in this paper. The FEM results for the novel device, illustrated in Figure 6b, demonstrated that peak displacement occurred at an excitation frequency of 77.1 kHz. On the other hand, the Finite Element Model of the control device exhibited peak displacement at the higher excitation frequency of 139 kHz.

In the subsequent phase of the Finite Element Modelling, the interaction of the novel device with incoming ultrasonic radiation was examined. This phase aimed to assess whether the pinned diaphragm design provided advantages for a PMUT when operating in ultrasonic radiation reception mode. The same FEM structure depicted in Figure 8b was employed again. However, in this instance, the diaphragm was not electrically excited. Instead, incident ultrasonic radiation at an acoustic pressure of 40 Pa was introduced into the model through the boundary marked in blue, thereby acoustically exciting the PMUT’s diaphragm. The results, including the resonant frequency and peak displacement achieved, are detailed in Table 1.

As shown in Table 1, the FEM results for acoustic excitation, indicate that the novel device is expected to resonate at a lower frequency than the control device. This is consistent with the results achieved from the electrical stimulation FEM. Additionally, Table 1 suggests that the novel device would exhibit a significant increase in peak displacement at resonance when acoustically excited. Since the displacement of the piezoelectric layer generates a voltage, this Finite Element Modelling indicates that the novel device is anticipated to present higher sensitivity to incoming ultrasonic radiation.

Once the optimised design was established, mask development was conducted following the PiezoMUMPS^TM^ design process, as described in the subsection below.

### 2.4. The Device Manufacturing Processes

The process flow steps that are required to produce the devices are shown in Table 2 below. This includes five masks through which the patterning, etching and deposition processing accomplished were conducted. The substrate used for this PiezoMUMPS^TM^ device was a 400 µm thick <100> oriented Silicon-on-Insulator (SOI) wafer. Before the processes outlined in Table 2 commenced, the wafer was annealed for 1 h in argon [17]. Following the conclusion of the annealing process, a thermal oxide layer, which was 0.2 µm thick, was grown over the substrate. Above this oxide layer, a positive photoresist coating was applied.

The first mask level that was designed was the PADOXIDE level mask. This mask was used to conduct the photolithographic patterning of the photoresist layer. These patterns were then used to wet etch the Pad Oxide layer; then, the photoresist layer was stripped. A process of reactive sputtering was next used to deposit the piezoelectric layer. For this reactive sputtering process, the next mask that was used was the PZFILM level mask, which was used to pattern the AlN, forming the piezoelectric layer.

The next step in the process was the creation of the metal structures through the Pad Metal Liftoff process. The metal structures were created through the beam evaporation process. The metal layer thus created is known as the PadMetal layer and consists of a metal stack with a 20 nm thick chromium layer underlying a 1 µm thick aluminium layer. As can be seen in Table 2, the PADMETAL mask used for this layer contained the intricate design required for the electrical stimulation circuit. The conducting tracks were 5 µm wide, with the returning electrical path passing through the doped silicon layer situated beneath the 0.5 µm thick AlN piezoelectric layer.

Following the build-up of all the layers making up the device, the next stage to proceed was the Silicon Patterning process, which was conducted through the use of Deep Reactive Ion Etching (DRIE). This process included patterning the entire suspended central structure that also comprised the suspending arms that held it in place. These features on the silicon were etched into the 10 µm thick phosphorus-doped silicon slab. The centrally suspended diaphragm of the novel device had a diameter of 410 µm. Each of the eighteen supporting arms measured 20 µm in width. A summary of the key design parameters for the PMUT device is presented in Table 3.

**Figure 9:**
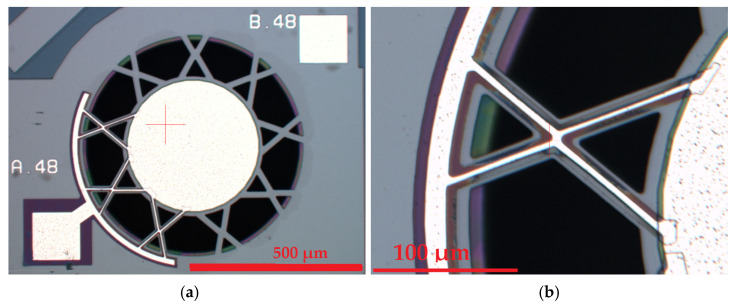
(**a**) Micrograph offering a frontal view of the novel device, highlighting the electrical conductors integrated within the suspending arms. (**b**) High magnification micrograph showing the detail of the holding arms [7].

Electrical connections to the metal electrode, located centrally at the top of the suspended diaphragm, were routed through six of the suspending arms, as illustrated in Figure 9a. Figure 9b shows a high magnification micrograph of the supporting arm structure with the electrical conductors passing over the SOI structure. This allows the user to view the critically small tolerances in play. In comparison, one can keep in mind that, on average, a human hair has a diameter ranging between 70 and 120 microns.

The control device was similarly produced following the same process steps outlined in Table 2. The next stage of the project was to experimentally confirm the predictions of the Finite Element Modelling by benchmarking the performance of the novel configuration, which was compared to the fully clamped device shown in Figure 2 that was used as the control device. The cavity diameter for both the novel and control devices was 650 µm.

## 3. Experimental Work

Next, the project entered the benchmarking experimental stage where the novel and control devices were tested side by side. Both devices had an air filled cavity and were deployed in liquid isopropanol coupling fluid. For the control device a top electrode which radially covered 66% of the AlN radius was used to excite the piezoelectric layer. The value of radial coverage was selected based on the literature review, which indicated that this value provides the best dynamic performance [18].

The prototype devices were subsequently subjected to experimental characterisation to validate the results obtained from the finite element models.

### 3.1. Ultrasonic Radiation Transmission: Resonant Frequency and Device Dynamics

The first experimental process that was conducted had the aim of determining the PMUTs’ resonant frequency and relevant dynamic parameters when being electrically excited, i.e., when operating in transmission mode.

**Figure 10:**
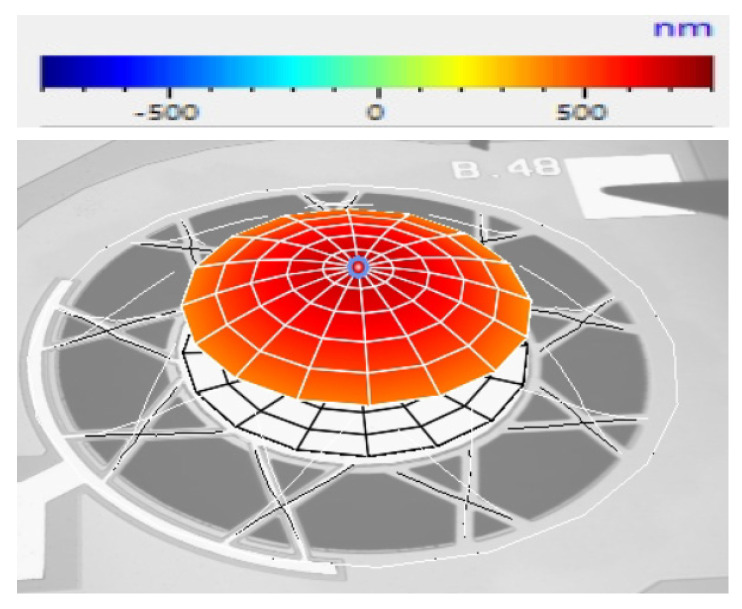
Laser vibrometer plot showing the peak displacement when the novel device was electrically excited by a sinusoidal signal having an amplitude of 14 V_p–p_ and a frequency of 69.38 kHz [7].

A sinusoidal electrical signal with a 14 V_p–p_ amplitude was used to excite the AlN piezoelectric layer of both the novel and control devices. The diaphragm’s movements during the excitation process were observed via a Polytec laser vibrometer. For the pinned diaphragm device, the resonant frequency was found to be 69.38 kHz and the peak PMUT diaphragm displacement at resonance can be seen in Figure 10.

The experimental value of the resonant frequency for the control PMUT was determined to be 119.53 kHz. As predicted by the FEM, it was, therefore, confirmed to be significantly higher than the resonant frequency achieved by the pinned diaphragm device. Table 4 outlines the dynamic parameters that were measured by the laser vibrometer for both the novel and control devices, at their respective point of resonance.

Table 4 indicates that, when electrically excited, apart from achieving a lower resonant frequency than that achieved by the control device, the novel device also exhibited a higher peak displacement. This peak displacement parameter indicated that the diaphragm of the novel device demonstrated a higher propensity to displace.

These experimental results were in line with the results achieved by the Finite Element Modelling outlined in the previous section.

### 3.2. Ultrasonic Radiation Detection: Resonant Frequency and Device Dynamics

The acoustic experimental process was conducted to study the devices’ dynamic performance when excited with an incident acoustic wave front. This was performed to experimentally establish the novel and control devices’ resonant frequency as well as their sensitivity when the devices were detecting incoming ultrasonic radiation.

**Figure 11:**
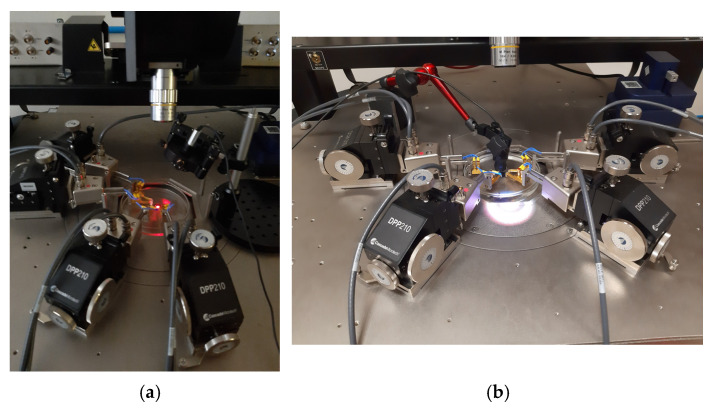
Experimental setup showing close up views of the probe station with close up views of (**a**) The laser diffraction experiment and (**b**) the acoustic testing setup showing the red supporting structure for the hydrophone/ultrasonic projector [7].

The ultrasonic testing process was conducted by setting up the devices on a probe station, which was specially configured to probe exposed semiconductor dies deployed in liquid coupling fluids. Views of the probe station are shown in Figure 11.

The close-up views of the probe station show the important components necessary to conduct the experimental process. Common to both Figure 11a,b are the microscope, fluid containment Petri dish and probes, which contact the die through the needles. The fluid containment Petri dish contained the coupling fluid and probed die.

Figure 11a further presents the laser equipment used for resonant point determination exercises. The laser equipment was from Thor Labs, USA. This setup was utilised to double check the resonant frequency point, which was established through the acoustic excitation process. The 532 nm wavelength CPS532-Collimated Laser-Diode Pumped (Thorlabs, Newton, NJ, USA) DPSS Laser Module that was used, produced a collimated 4.5 mW round beam, which was aimed at the PMUT’s reflective upper electrode. The reflected laser light was projected onto a screen and the image was inspected. Through close inspection of the projected image, the onset of resonance was detected. The reason being that fringing on the reflected image was observed when the PMUT was vibrating at its point of resonance. This phenomenon occurred due to diffraction of the laser beam, which happened due to the high amplitude of the PMUT’s displacement at the point of resonance.

On the other hand, Figure 11b presents the hydrophone setup. At the heart of this setup was the Benthowave BII-7001 (Benthowave, Collingwood, ON, Canada), a combined hydrophone and ultrasonic projector made in Canada. The projector’s acoustic centre was submerged in the coupling fluid, with the tip being displaced 40 mm laterally and 4 mm above the probed PMUT.

For the acoustic excitation process, the ultrasonic projector was configured to transmit ultrasonic radiation at a source pressure of 40 Pa, spanning a frequency range between 60 kHz and 140 kHz. This low-pressure level was deliberately chosen so as to evaluate the ultrasonic reception capabilities of the PMUTs under conditions of weak incoming radiation. The reason for this is the determination of the maximum separation distance possible between the sensory devices.

**Figure 12:**
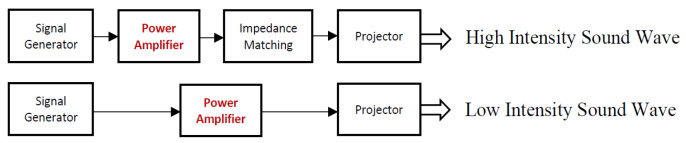
System connection block diagram for the Ultrasonic Projector [19].

Figure 12 shows the block diagram of the system used to control the output of the Ultrasonic Projector. For this project, low-intensity sound waves were used and therefore impedance matching was not utilised. The pressure was kept constant throughout the frequency sweep by conducting adjustments to the power amplifier’s DC voltage input at every frequency step. This was carried out to keep the Transmitted Voltage Response (TVR) constant for all the frequency spectrum.

Voltage measurements across the piezoelectric layer were recorded as the frequency was incrementally swept across the entire range. The frequency was increased in steps of 500 Hz. The resonant point was identified at the frequency where the peak voltage was observed.

**Figure 13:**
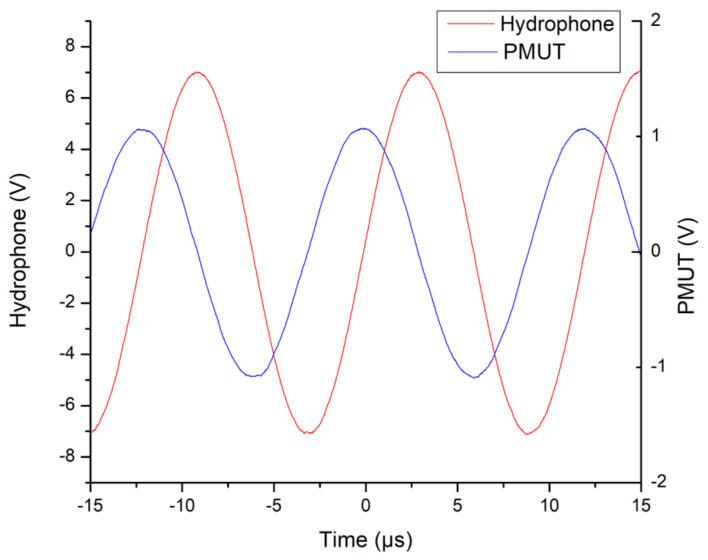
Graph showing the voltage measured directly across the novel 650 µm diameter PMUT (blue) and the signal received by the hydrophone/ultrasonic projector’s power amplifier from the signal generator (red). The frequency is 82.99 kHz.

It was observed that, for the novel device, an electrical signal across the piezoelectric layer of the device can be measured at various frequencies of incoming ultrasonic radiation. The most significant response occurred at an incident ultrasonic frequency of 82.99 kHz, where an electrical signal with a peak voltage of 2.183 V_p–p_ was measured across the piezoelectric layer of the novel PMUT. Figure 13 presents the voltage across the piezoelectric layer of the novel PMUT plotted together with the driving signal being fed into the ultrasonic projector’s high voltage source at a frequency of 82.99 kHz.

The novel device’s piezoelectric layer presented significant output voltages at other excitation frequencies, the most significant of which occurred at 94.2 kHz followed by 85.21 kHz when signals having amplitudes of 1.971 V_p–p_ and 1.3 V_p–p_, respectively, were measured across the piezoelectric layer of the novel PMUT.

By contrast, when the control device was subjected to acoustic radiation across the same frequency range as the novel device, it did not exhibit any measurable voltage across its piezoelectric layer. This result indicates that the 650 µm diameter control device was not easily excited acoustically at any frequency within the scanned range, when the ultrasonic radiation was incoming at a low pressure of 40 Pa. As stated, this value of ultrasonic pressure was used for both the novel and control devices.

To conclude the acoustic reception benchmarking exercise for the novel device, its performance was compared with that of a 700 µm diameter control device. The larger diameter was chosen because the resonant frequency of the 700 µm device is lower and thus closer to that of the novel device, as per Equation (1). The 700 µm clamped diaphragm control device shared the same geometry as the 650 µm PMUT, with the only difference being the larger diaphragm diameter.

**Figure 14:**
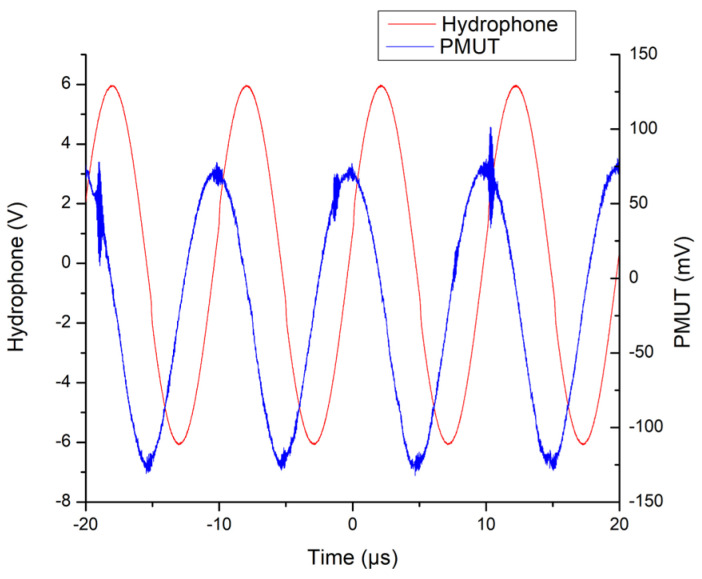
Graph showing the voltage measured directly across the 700 µm diameter PMUT (blue) and the signal received by the hydrophone/ultrasonic projector’s power amplifier from the signal generator (red). The frequency is 99 kHz.

The 700 µm diameter control device was set up using the same equipment as the other two devices and subjected to the same 40 Pa ultrasonic frequency sweep. Compared with the 650 µm diameter control device, the 700 µm diameter control device was successfully acoustically excited and produced a voltage across the piezoelectric layer when stimulated at a frequency of 99 kHz. The voltage across the piezoelectric layer overlaid over the signal that is fed into the Hydrophone/Ultrasonic Projector’s power amplifier can be seen in Figure 14.

As can be seen from Figure 14, the V_p–p_ for the 700 µm diameter PMUT was 234 mV. This is more than nine times lower than that measured across the novel device.

## 4. Discussion

The FEM and experimental results discussed in this paper demonstrate that the novel pinned diaphragm design effectively lowered the PMUT’s resonant frequency. Additionally, the novel design significantly enhanced the PMUT’s ability to detect ultrasonic radiation, showing considerably greater sensitivity to incoming radiation compared with a circular clamped diaphragm device of the same diameter. The acoustic benchmarking exercise with the 700 µm diameter clamped diaphragm control device indicated that the 650 µm diameter novel device is an even more sensitive receiver of ultrasonic radiation than the larger 700 µm clamped diaphragm device, by a factor of nine.

## 5. Conclusions

This project aimed to develop a PMUT device operating at a lower resonant frequency with enhanced ultrasonic reception capabilities. The work conducted and presented in this paper included Finite Element Modelling and device fabrication, as well as the experimental work necessary to benchmark and test the device. The results achieved by the novel device in both the Finite Element Modelling and the experimental processes have shown that the aims were indeed achieved.

These results have important implications for various applications, including medical imaging and non-destructive testing, where precise and reliable ultrasonic detection is crucial. Future work should focus on further refining the device design and exploring its integration into commercial systems to fully achieve its potential benefits. The novel device geometry presented in this paper can be an important tool for designing compact and sensitive ultrasonic PMUT receivers. It is also important to note that potential applications for the novel design are not limited to Structural Health Monitoring. There can also be vast application possibilities in other areas which utilise ultrasonic technology such as the biomedical field.

Further research work would be required to further improve this design. One potential improvement can be enhancing the design to prevent liquid migration from the coupling region to the cavity region. The novel device’s unclamped diaphragm has gaps that may allow liquid to migrate when the PMUT is oriented with the cavity region below the coupling region. This liquid infiltration displaces the air within the cavity region, leading to changes in the PMUT’s dynamic parameters, such as its resonant frequency.

At the current stage of development, to prevent liquid ingress into the cavity, the novel device is deployed in an inverted orientation, as depicted in Figure 1. In this configuration, the diaphragm remains suspended above the coupling fluid. This setup could be suitable for Structural Health Monitoring applications, where the sensor systems, including the PMUTs, remain stationary and embedded within the concrete structure.

However, this method may be inadequate for applications in the biomedical domain (and others), where sensor systems require mobility. Therefore, further research is necessary to address this limitation. This could involve developing fluids with enhanced surface tension properties and improving arm designs, such as incorporating micro hairs in the arms to enhance surface tension effects and prevent fluid passage.

**Figure 15:**
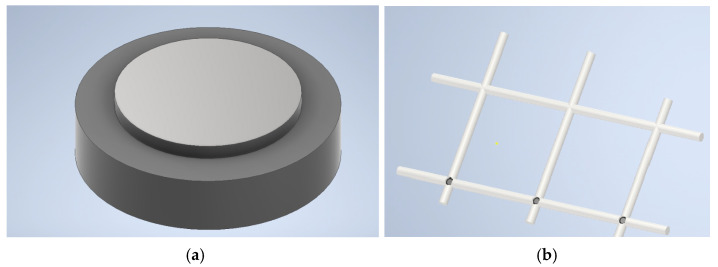
Conceptual drawings showing (**a**) the capsule containing the sensory system including the PMUT and (**b**) capsules embedded in a grid structure [7].

Further research is also ongoing in the area of the encapsulation which is necessary to protect the fragile PMUT from the harsh environment of the concrete pore solution. While this will be the subject of future work, a conceptual drawing is shown in Figure 15. The capsules are designed to be produced from low-density polyethylene with a diameter of 10 mm and a wall thickness of 1 mm. The interior of the capsule is filled with isopropanol to acoustically couple the PMUT to the capsule’s skin. On the outside of the capsule there is a layer of glycerine (or similar substance) to complete the coupling between the capsule’s outer skin and the concrete.

To make it easy for construction workers to place the devices at the correct position within the structure, the capsules can be pre-positioned at their manufacturing site by being embedded in a grid structure as shown in Figure 15b. The grids are then placed in the concrete that can be either an in situ pour or precast reinforced concrete products. Such a system allows rapid installation of the devices in a way that protects the delicate PMUTs.

The novel PMUTs underwent lab testing for reliability, where they were electrically stimulated at their resonant frequency for five hours, surpassing the expected operational period for a deployed PMUT device. Throughout testing, the devices were periodically examined for signs of stress or degradation. Microscopic evaluation revealed no physical degradation.

In conclusion, it can therefore be stated that the paper successfully demonstrated that by utilising a pinned diaphragm structure, it was possible to achieve a significant reduction in resonant frequency while simultaneously improving ultrasonic reception capabilities. With further research, a class of pinned diaphragm PMUTs with enhanced capabilities can be developed, catering for different ultrasonic frequency requirements.

## Figures and Tables

**Table 1:** FEM results for novel and control devices operating in ultrasonic reception mode. Both with a diameter of 650 µm.

	Resonant Frequency [Hz]	Peak Displacement at Diaphragm’s Midpoint [µm]
**Novel Device**	0.99 × 10^5^	0.25
**Control Device**	1.505 × 10^5^	0.049

**Table 2:** Process steps and mask levels required to produce a PiezoMUMPS^TM^ device [17].

Mask Level~Process	Mask	Section through Device
PADOXIDE~Thermal Oxide	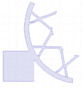	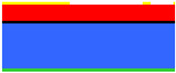
PZFILM~Film Liftoff	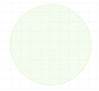	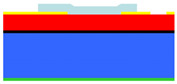
PADMETAL~Padmetal Liftoff	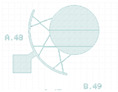	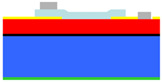
SOI~Silicon Patterning	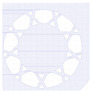	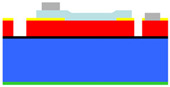
TRENCH~Substrate Patterning	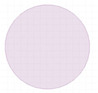	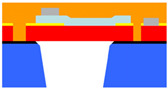

**Table 3:** Key design parameters for the novel PMUT device.

	Cavity Diameter [µm]	Suspended Diaphragm Diameter [µm]	Number of Supporting Arms	Width of Arms [µm]	Electrode Diameter [µm]
Dimensions	650	410	18	20	400

**Table 4:** Dynamic parameters of novel and control PMUT devices as measured by laser vibrometry when excited by a 14 V_p–p_ sinusoidal electrical signal.

Device Description	Control Device [Ø 650 µm]	Novel Device [Ø 650 µm]
Peak Displacement [µm]	0.486	0.528
Peak Velocity [m/s]	0.3659	0.233
Peak Acceleration [km/s^2^]	274.797	102.26
Resonant Frequency [kHz]	119.53	69.38

## Data Availability

The data supporting this study’s findings are available from the corresponding authors upon reasonable request.
